# The Relationship Between Prematurity and Mode of Delivery with Disorders of Gut–Brain Interaction in Children

**DOI:** 10.3390/children12060799

**Published:** 2025-06-18

**Authors:** Carlos Alberto Velasco-Benitez, Daniela Alejandra Velasco-Suarez, Natalia Palma, Samantha Arrizabalo, Miguel Saps

**Affiliations:** 1 Department of Pediatrics, Universidad del Valle, Cali 76001, Colombia; carlos.velasco@correounivalle.edu.co (C.A.V.-B.);; 2 School of Medicine, Peruvian University of Applied Sciences, Lima 15023, Peru; natpalmayri.97@gmail.com; 3 Department of Pediatrics, Division of Gastroenterology, Hepatology and Nutrition, Miller School of Medicine, University of Miami, Miami, FL 33136, USA; msaps@med.miami.edu

**Keywords:** disorders of gut–brain interaction, cesarean delivery, vaginal delivery, prematurity, infants, school-age infants, functional constipation

## Abstract

**Background:** Disorders of gut–brain interaction (DGBI) are multifactorial. Early-life events are proposed as factors involved in their etiopathogenesis. The relationship between mode of delivery, prematurity, and DGBI development remains unclear. This study examines whether cesarean delivery and prematurity contribute to early childhood DGBI. **Methods:** Caregivers of children aged 1 month to 4 years from four Colombian cities participated in a cross-sectional study. Pediatricians completed the Spanish-validated Questionnaire of Pediatric Gastrointestinal Symptoms Rome IV (QPGS-IV). Data of children born by cesarean delivery and prematurity were compared with controls. Categorical data were analyzed using Fisher’s exact test, and odds ratios (ORs) with 95% confidence intervals (CIs). **Results**: DGBIs were identified in 26.6% of children, with functional constipation (FC) being the most prevalent (22.3%). Among children born via cesarean section (54.3%), 30.4% of them had a DGBI (OR = 1.54, 95% CI = 1.20–1.96, *p* = 0.00), and 26.3% had FC (OR = 1.67, 95% CI = 1.29–2.18, *p* = 0.00). Prematurity was observed in 12.6% of children and was associated with a higher prevalence of DGBI (35.7%, (OR = 1.64, 95% CI = 1.16–2.29, *p* = 0.00), with FC affecting 30.8% (OR = 1.66, 95% CI = 1.16–2.35, *p* = 0.00). **Conclusions**: Cesarean delivery and prematurity were found to be associated with DGBI in early childhood, particularly FC. These findings highlight the need for further research to explore potential mechanisms and confirm these associations.

## 1. Introduction

Disorders of gut–brain interaction (DGBI), as defined by the Rome IV criteria, are characterized by chronic gastrointestinal symptoms without identifiable structural or biochemical abnormalities to explain patient symptoms [1]. They are highly prevalent worldwide, affecting 25% of children and 41.4% of adults [2,3]. The biopsychosocial model links genetic susceptibility, gut microbiota imbalances, and psychosocial factors to the development of DGBI [4]. Early-life events, such as the method of delivery and gestational age at birth, have been suggested as contributing factors in some studies [5], but their role remains controversial [6,7,8]. While some studies point to a clear association with the development of DGBI in school-age children [5] and adults [6,8], others yield conflicting results [7,9], highlighting the need for further investigation.

Early gut colonization plays a crucial role in shaping long-term health and disease outcomes, although the exact causes remain unclear [10]. While the fetal microbiota was once believed to remain sterile in the uterine environment [11], recent studies suggest that microbial colonization may begin in utero [12,13]. In preterm infants, gut microbiota development differs significantly from that of term infants, likely influenced by higher rates of cesarean delivery [14], antibiotic use, necrotizing enterocolitis and admission to neonatal intensive care [5].

The mode of delivery significantly influences the composition of an infant’s gut microbiome [15]. Vaginally delivered infants typically acquire microbial communities resembling their mother’s vaginal microbiota, which includes beneficial microorganisms. In contrast, cesarean-delivered infants are more likely to develop a microbiome dominated by skin flora or potentially pathogenic bacteria [10,16], which may increase the risk of DGBIs during both childhood and adulthood [17].

Studies in both adults and children with DGBI, such as irritable bowel syndrome (IBS), have shown differences in gut microbiota profiles compared to controls [18]. Notably, research in pediatric patients identified a higher relative abundance of the genus *Blautia* and a lower abundance of *Bacteroides* in their gut microbiota [19]. These microbial alterations may play a role in the pathophysiology of DGBI. It is unclear if early-life microbial exposures contribute to shaping the future microbiota of this group of children.

Data on the relationship between mode of delivery, prematurity, and the development of DGBI in children remain limited and contradictory. Most studies have been conducted in Europe [6,8], with none specifically focusing on infants and preschool-aged children in Latin America. We hypothesize that cesarean delivery and prematurity are associated with a higher prevalence of DGBI in infants and preschool-aged children of Colombia. This study aims to assess whether cesarean delivery and prematurity contribute to the development of DGBI in early childhood.

## 2. Materials and Methods

### 2.1. Participant Recruitment

Caregivers of children aged 1 month to 4 years—categorized into two groups (1–12 months and 1–4 years)— who attended pediatric outpatient clinics at public and private hospitals in four geographically dispersed Colombian cities (Florencia, Sotavento, Cali, and Bogotá), were invited to participate in a cross-sectional study.

Inclusion criteria: children aged between 1 month and 4 years of age. Exclusion criteria: infants with a history of organic diseases.

Prior to participation, caregivers were informed about the study and provided consent.

### 2.2. Data Collection

Data were obtained by interviewing the parents. Before completing the questionnaires, a member of the research team explained to the practitioner how to complete the validated Spanish version of the Questionnaire of Pediatric Gastrointestinal Symptoms Rome IV (QPGS-IV) [20]. During the child’s appointment, the clinician completed the questionnaire and clarified any questions the caregivers had. Additionally, two of the study authors (Dr. Miguel Saps and Dr. Carlos Alberto Velasco-Benítez), both pediatric gastroenterologists, were involved in the design and interpretation of the study to ensure the clinical relevance and accuracy of the collected data.

### 2.3. Statistical Analysis

Demographic data were analyzed using measures of central tendency, including mean ± standard deviation (SD). We assessed the relationship between prematurity, cesarean delivery, and the development of DGBI by comparing the prevalence of DGBI in children with and without these factors. Univariate analysis was conducted to calculate odds ratios (ORs) with 95% confidence intervals (95% CIs). The significance level was set at *p* < 0.05. Data were analyzed using Stata 10 software (StataCorp, College Station, TX, USA).

### 2.4. Ethical Approval

The study was approved by the Ethics Committee of the Hospital Universitario del Valle “Evaristo García” in Cali, Colombia (023-2019, 28 March 2019).

## 3. Results

**Figure 1:**
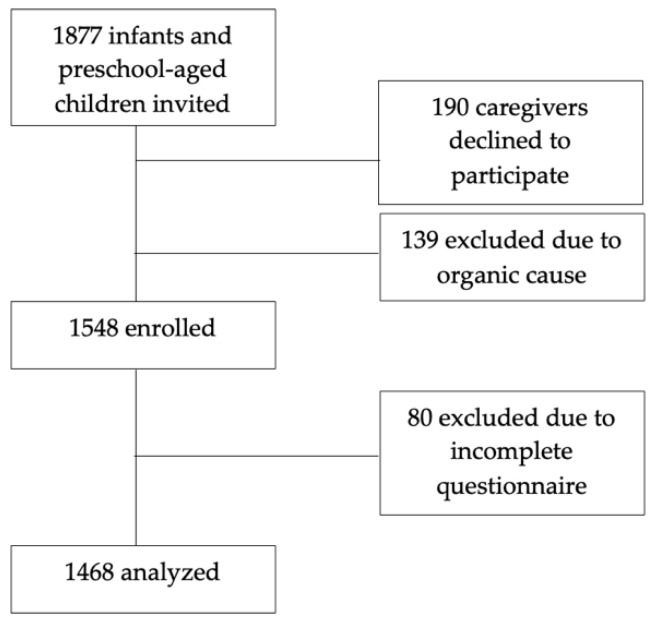
Flowchart of study enrollment for infants and preschool-aged children.

A total of 1877 caregivers were invited to participate in the study. Of these, 409 were not enrolled/excluded: 190 because caregivers declined participation, 139 due to organic diseases, and 80 due to incomplete questionnaires. Data from the remaining 1468 infants and preschool-aged children (94.8%) were included in the analysis (Figure 1). Most children were identified as white (36%) or with a mixed (35.8%) ethnic background and 50.7% were male. The mean age of our sample was 24.2 ± 15 months (Table 1).

In total, 390 (26.6%) infants and preschool-aged children were diagnosed with at least one DGBI. The most prevalent disorder was functional constipation (FC) (22.3%) (Table 2), with 56 (10.9%) cases in children under 1 year of age and 272 (28.5%) in those aged 1 to 4 years (Table 3). Infant colic was the second most common diagnosis, observed in 4% of infants (n = 126) (Table 2).

Among the total number of infants and preschool-aged children, 797 (54.3%) were born via cesarean section, while 45.7% were delivered vaginally. Among infants born by cesarean section, 242 (30.4%) were diagnosed with DGBI (OR = 1.54, 95% CI = 1.20–1.96, *p* = 0.00). Of these, 210 (26.3%) were diagnosed with FC (OR = 1.67, 95% CI = 1.29–2.18, *p* = 0.00). The sample size for other disorders was too small to allow for appropriate analysis.

In terms of gestational age, 1283 infants (87.4%) were born at term, while 185 (12.6%) were preterm. In total, 66 (35.7%) infants and preschool-aged children were preterm and diagnosed with DGBI (OR = 1.64, 95% CI = 1.16–2.29, *p* = 0.00). Fifty-seven (30.8%) prematurely born children were diagnosed with FC (OR = 1.66, 95% CI = 1.16–2.35, *p* = 0.00). The sample size for other disorders was too small to allow for an appropriate analysis.

A total of 138 (17.3%) infants and preschool-aged children were born both prematurely and via cesarean section. Among them, 55 (40%) were diagnosed with DGBI (OR = 1.67, 95% CI = 1.11–2.48, *p* = 0.00), and 47 (34.1%) were diagnosed with FC (OR = 1.57, 95% CI = 1.03–2.36, *p* = 0.02).

## 4. Discussion

This study examined whether cesarean delivery and prematurity are associated with the development of DGBI in early childhood. Our findings suggest that children born either via cesarean section or prematurely have a higher likelihood of being diagnosed with DGBI compared to those born vaginally and at term. Moreover, children born with both risk factors were also at higher risk of DGBI.

Current research on these risk factors remains limited and sometimes contradictory [21,22]. Animal models have shown that perinatal stressors are associated with disruptions in gut physiological processes [23]. Notably, Kannampalli et al. (2014) [24] showed that administering probiotics and/or prebiotics modulated colonic sensitivity in neonatal rats, highlighting how early-life alterations in the microbiome can contribute to the development of visceral hyperalgesia. Preterm infants, especially those born at earlier gestational weeks, are more likely to endure physical and psychological stressors. Preterm infants are often separated from their mother and exposed to painful stimuli due to medical procedures in neonatal intensive care. Additionally, they are more likely to have health complications and to receive antibiotics that may increase the risk of DGBI [25]. Preterm infants exhibit lower microbial diversity and a distinct colonization by facultative anaerobic bacteria compared to term infants [26]. Recent research has highlighted that this dysbiosis, characterized by a reduced microbiome diversity, may play a significant role in the physiopathology of disorders such as infant colic and FC [27]. In line with these findings, our study revealed that 35.7% of children born prematurely develop DGBI in early childhood, with 86.4% of them being diagnosed with FC. This high prevalence of constipation may partly reflect the broader diagnostic window of FC, which can be identified from infancy through 4 years of age, whereas other DGBIs are diagnosed only within the first months of life. Our study did not investigate which preterm infants were admitted to the intensive care unit or conduct microbiome analysis, both of which could provide further insights into the underlying mechanisms of these associations.

Previous studies on the possible effect of mode of delivery have shown some contradictory results. Two large Swedish cohort studies have investigated the relation between cesarean section and the prevalence of DGBI. Waehrens et al. (2017) [8] found a higher likelihood of IBS in young adults born by cesarean section. In contrast, Olén et al. (2018) [6] concluded that cesarean delivery was exclusively associated with an increased risk of IBS in women. The result of our study builds on the research of these previous studies. We found that one in three children born via cesarean delivery developed DGBI in early childhood. FC was significantly associated with this form of delivery. As the design of our study did not include the analysis of microbiota, we can only hypothesize about its possible effect. It is possible that dysbiosis associated with cesarean section could explain this association [15,28,29].

This is the first study to examine the correlation between prematurity, mode of delivery, and DGBI in children aged 1 to 4 years, expanding on our previous research in older children (10 to 18 years) [7]. While other studies have primarily focused on adults with IBS, our findings provide potentially valuable insights into early-life risk factors for DGBI. This study can potentially help fill the gap of knowledge of the time period between birth and the later age in which other studies have focused. One could hypothesize that if changes in microbiome influenced the development of DGBI at an older age, some changes could already be seen in young children. Our previous study failed to find a relationship between the same factors and DGBI in older children. If the results of both of our studies are confirmed, it could suggest that the effect of type of delivery and prematurity is lost over time. This could provide a potential explanation for the lack of effect seen in some adult studies [6,9] that have investigated these same factors.

Limitations of our study include the sample size that restricted the ability to analyze the likelihood of each individual disorder, precluding statistical analysis for less common disorders. Future studies with pooled data and larger, more diverse, and longitudinal cohorts are needed to clarify these associations and address potential confounding and enable a more comprehensive understanding of this possible relationship. Although we cannot assure that our results can be generalized to other populations, we found a prevalence of DGBIs similar to worldwide estimates [3,30,31]. Additionally, selection bias may be present, as the study population consisted of children who visited pediatricians, and may not reflect the reality of all children of the same age. Our data were based solely on parent-reported information from interviews and the QPGS-IV, a validated tool for diagnosing DGBIs. However, diagnoses were not confirmed clinically or through medical records, which may affect accuracy. Variables such as prematurity and delivery mode were also subject to recall bias. Future studies should confirm these data using medical records to enhance accuracy. Moreover, infant feeding practices (e.g., breastfeeding vs. formula feeding), which can significantly influence gut microbiota development and may differ between preterm and term infants, were not collected and could represent an unmeasured confounding factor. Furthermore, future research should incorporate detailed microbiome analyses to better characterize its composition and strengthen the biological plausibility of the observed associations in children born via cesarean or vaginal delivery, across preterm, term, and post-term births. Such studies would provide deeper insights into the impact of early-life factors on gut health and the development of DGBI.

## 5. Conclusions

Our findings suggest that cesarean delivery and prematurity are associated with an increased likelihood of DGBIs in early childhood. Further research is needed to validate these associations and investigate the role of microbiota, along with other contributing factors involved in DGBI development.

## Figures and Tables

**Table 1:** Demographic characteristics (n = 1468).

Demographics	n	%
**Age (months)**		
X ± SD	24.2 ± 15	
Range	1–53	
**Age groups**		
1–12 months	515	35.1
1–4 years	953	64.9
**Sex**		
Female	724	49.3
Male	744	50.7
**Race**		
White	528	36
Mixed ethnic background	525	35.8
Indigenous	339	23.1
Black	76	5.2
**City**		
Florencia	702	47.8
Sotavento	310	21.1
Cali	301	20.5
Bogota	155	10.6

**Table 2:** DGBI diagnoses among infants and preschool-aged children (n = 1468).

	Children with DGBI (n = 390)		All Children (n = 1468)
Functional constipation	328	84.1%	22.3%
Infant colic * (**n = 126**)	5	1.3%	4%
Infant regurgitation ** (**n = 515**)	16	4.1%	3.1%
Cyclic vomiting syndrome	32	8.2%	2.2%
Infant dyschezia *** (**n = 253**)	3	0.8%	1.2%
Rumination syndrome	3	0.8%	0.2%
Functional diarrhea	3	0.8%	0.2%

* Infants < 5 months; ** infants < 12 months; *** infants < 9 months.

**Table 3:** Prevalence of DGBI in infants (n = 515) and preschool-aged children (n = 953).

	Infants 1–12 Months n = 515 (%)	Children 1–4 Years n = 953 (%)	Total n = 1468 (%)
X +/− SD	8.3 +/− 3.9	2.7 +/− 0.9	
Functional constipation	56 (10.9%)	272 (28.5%)	328 (22.3%)
Infant colic	5 (4%)	N/A	5 (0.3%)
Infant regurgitation	16 (3.1%)	N/A	16 (1%)
Cyclic vomiting syndrome	0	32 (3.4%)	32 (2.2%)
Infant dyschezia	3 (1.2%)	N/A	3 (0.2%)
Rumination syndrome	0	3 (0.3%)	3 (0.2%)
Functional diarrhea	0	3 (0.3%)	3 (0.2%)

## Data Availability

The data presented in this study are available on request from the corresponding author. The data are not publicly available due to privacy and ethical restrictions.

## Abbreviations

The following abbreviations are used in this manuscript:

DGBI	Disorders of gut–brain interaction
FC	Functional constipation
IBS	Irritable bowel syndrome
QPGS-IV	Questionnaire of Pediatric Gastrointestinal Symptoms Rome IV
